# Rapid Changes in Cardiac Myofilament Function following the Acute Activation of Estrogen Receptor-Alpha

**DOI:** 10.1371/journal.pone.0041076

**Published:** 2012-07-30

**Authors:** Justyna Kulpa, Nirmala Chinnappareddy, W. Glen Pyle

**Affiliations:** 1 Cardiovascular Research Group, Department of Biomedical Sciences, Ontario Veterinary College, University of Guelph, Guelph, Ontario, Canada; 2 Biophysics Interdepartmental Group, University of Guelph, Guelph, Ontario, Canada; Ohio State University, United States of America

## Abstract

Estrogens have well-recognized and complex cardiovascular effects, including altering myocardial contractility through changes in myofilament function. The presence of multiple estrogen receptor (ER) isoforms in the heart may explain some discrepant findings about the cardiac effects of estrogens. Most studies examining the impact of estrogens on the heart have focused on chronic changes in estrogen levels, and have not investigated rapid, non-genomic pathways. The first objective of this study was to determine how acute activation of ERα impacts cardiac myofilaments. Nongenomic myocardial estrogen signaling is associated with the activation of a variety of signaling pathways. p38 MAPK has been implicated in acute ER signaling in the heart, and is known to affect myofilament function. Thus, the second objective of this study was to determine if acute ERα activation mediates its myofilament effects through p38 MAPK recruitment. Hearts from female C57Bl/6 mice were perfused with the ERα agonist PPT and myofilaments isolated. Activation of ERα depressed actomyosin MgATPase activity and decreased myofilament calcium sensitivity. Inhibition of p38 MAPK attenuated the myofilament effects of ERα activation. ERα stimulation did not affect global myofilament protein phosphorylation, but troponin I phosphorylation at the putative PKA phosphorylation sites was decreased. Changes in myofilament activation did not translate into alterations in whole heart function. The present study provides evidence supporting rapid, non-genomic changes in cardiac myofilament function following acute ERα stimulation mediated by the p38 MAPK pathway.

## Introduction

Although premenopausal women are protected from cardiovascular morbidity as compared with age-matched men, the risk of heart disease increases in women following menopause [1]. This observation has lead to the hypotheses that endogenous estrogens are cardioprotective, and the replacement of estrogens in postmenopausal women should decrease the occurrence of heart disease in this population. Whereas this theory has been supported by observational [2] and animal studies [3], [4], [5], recent large-scale clinical trials have failed to demonstrate cardioprotection with postmenopausal hormone replacement therapy (HRT). In fact, some of these trials, including the Heart and Estrogen/progestin Replacement Study (HERS) and the estrogen + progestin arm of the Women’s Health Initiative (WHI), have reported adverse cardiovascular outcomes in subjects given supplemental estrogen [6], [7], [8]. Although a number of theories have arisen as to why these trials failed to show estrogen-mediated cardioprotection, including the age of HRT onset and the formulation and duration of the pharmaceuticals involved [9], the discrepant results have prompted interest into the molecular mechanisms of estrogen action in the heart.

Estrogen action can be mediated through binding to one of three known estrogen receptors (ER). It has been shown that ERα, ERβ and GPR30 are expressed in adult cardiac myocytes [10], [11], [12]. Estrogen can initiate intracellular responses through the classical genomic pathway, or through rapid, nongenomic pathways. The latter pathway is mediated through membrane-bound ER and mediated through several intracellular signaling pathways. The presence of ERβ at the cardiac myocyte membrane has not been confirmed [11]. However, a number of studies have identified sarcolemmal ERα [11], [13] and several reports have suggested that ERα activation is critical in protecting the heart against a variety of stressors [14], [15]. Despite its known cardioprotective effects, the intracellular mechanisms by which ERα regulates myocardial function has not been fully elucidated. p38 MAPK has been implicated in E2-mediated signaling in the heart and the cardioprotective effects of ERα activation [16], [17], but no studies have examined the links between the ERα and p38 MAPK under physiological conditions.

**Figure 1:**
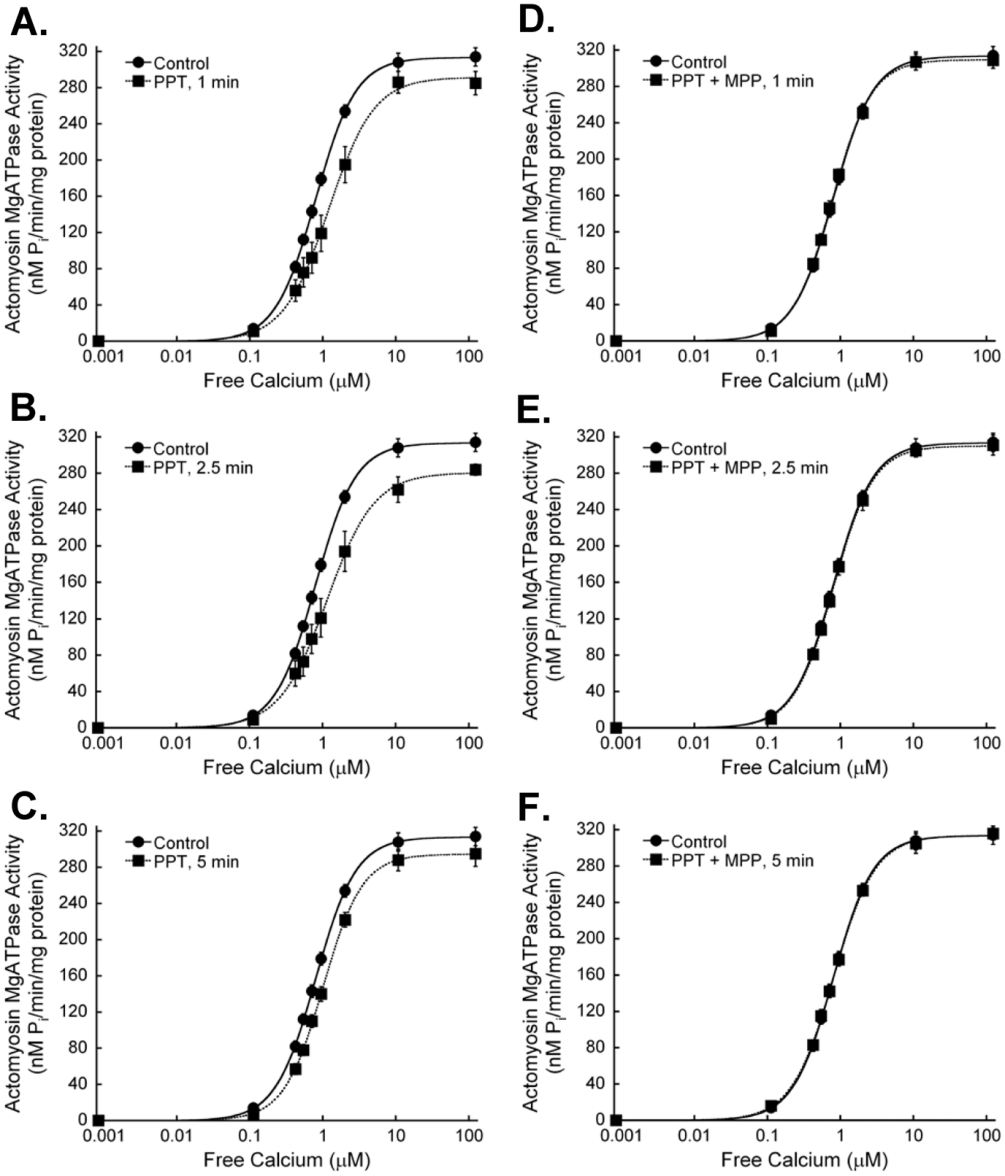
The estrogen receptor-α agonist PPT decreases cardiac myofilament activation. Myocardium was treated with the estrogen receptor-α agonist PPT (100 nM) for 1 (A) (N = 9), 2.5 (B) (N = 9), or 5 min (C) (N = 22). Untreated controls showed no differences at any time, and are combined as a single group in each graph (N = 23). Maximum actomyosin MgATPase activity tended to decrease following PPT treatment, but did not reach statistical significance. Actomyosin MgATPase activity at submaximal calcium levels (<10 µM free calcium) was decreased with estrogen receptor-α activation. EC_50_ values were increased, demonstrating a decrease in myofilament calcium sensitivity. The estrogen receptor-α antagonist MPP (1 µM) blocked the effects of 1 (D) (N = 3), 2.5 (E) (N = 3), and 5 min (N = 5) (F) PPT.

Cardiac myofilaments constitute the central contractile apparatus in the heart, and changes to their biochemical properties, notably their interaction with calcium, can alter the mechanical properties of the whole heart. Chronic E2 withdrawal following ovariectomy results in hypersensitivity of myofilaments to calcium [18], and this phenotype can be reversed through E2 replacement [19]. While these studies demonstrate how chronic changes in E2 levels can impact myofilament function, how cardiac myofilaments are affected by acute ER activation has not previously been investigated.

The purpose of the current study was to determine whether the acute and specific activation of ERα results in changes in myofilament function. Furthermore, we sought to determine if p38 MAPK is involved in the rapid activation of ERα in cardiac myocytes, and if it mediates the effects of ERα on cardiac myofilaments.

**Table 1:** Effects of ERα activation on cardiac myofilament function.

		PPT (100 nM)		
	Control	1 min	2.5 min	5 min
Maximum ATPase	314±10	282±11	280±10	295±14
EC_50_	0.81±0.03	1.37±0.10*	1.61±0.11*	0.99±0.05*
Hill	1.67±0.08	1.63±0.09	1.51±0.11	1.68±0.06

**Key:** Maximum ATPase, actomyosin MgATPase at maximally activating calcium, nM Pi/min/mg protein; EC_50_, free calcium (µM) required to activate actomyosin MgATPase at 50% of maximum; Hill, Hill coefficient. *P<0.05 vs Control.

**Figure 2:**
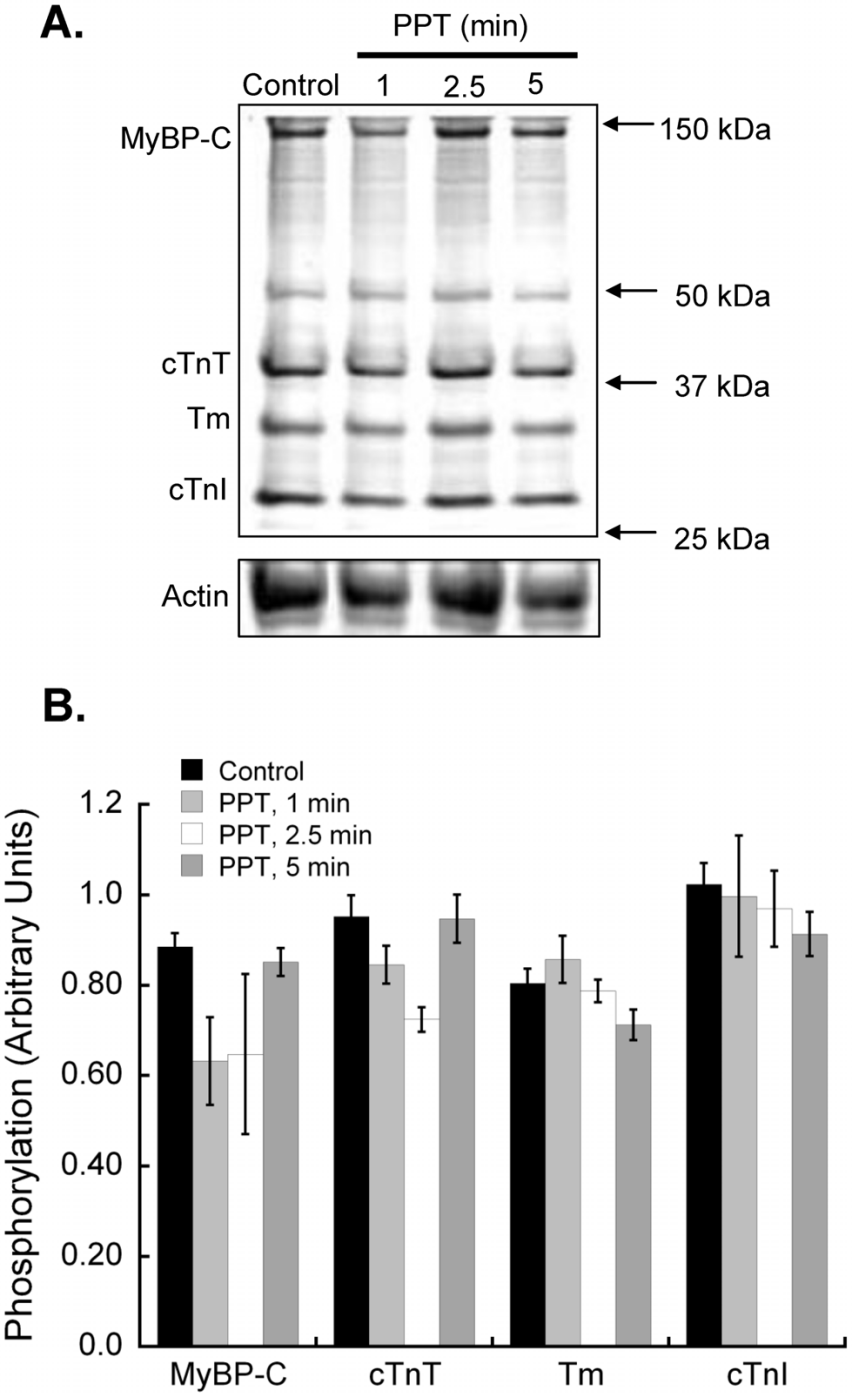
Activation of estrogen receptor-α does not alter total phosphorylation of myofilament proteins. Hearts were perfused with PPT (100 nM) for up to 5 min and cardiac myofilament isolated. **A.** Isolated cardiac myofilaments were separated by SDS-PAGE on a 12% gel. Gels were stained with Pro-Q Diamond Phosphoprotein gel stain to assess global phosphorylation. Equal protein loading was confirmed by Coomassie staining. The actin band from Coomassie stained gels is shown as representative of protein loading. **B.** Analysis of gels for global phosphorylation of myosin-binding protein C (MyBP-C), troponin T (cTnT), tropomyosin (Tm), and troponin I (cTnI) revealed no significant changes in phosphorylation status after activation of estrogen receptor-α for 1, 2.5 or 5 minutes with 100 nM PPT. N = 5 for all groups.

**Figure 3:**
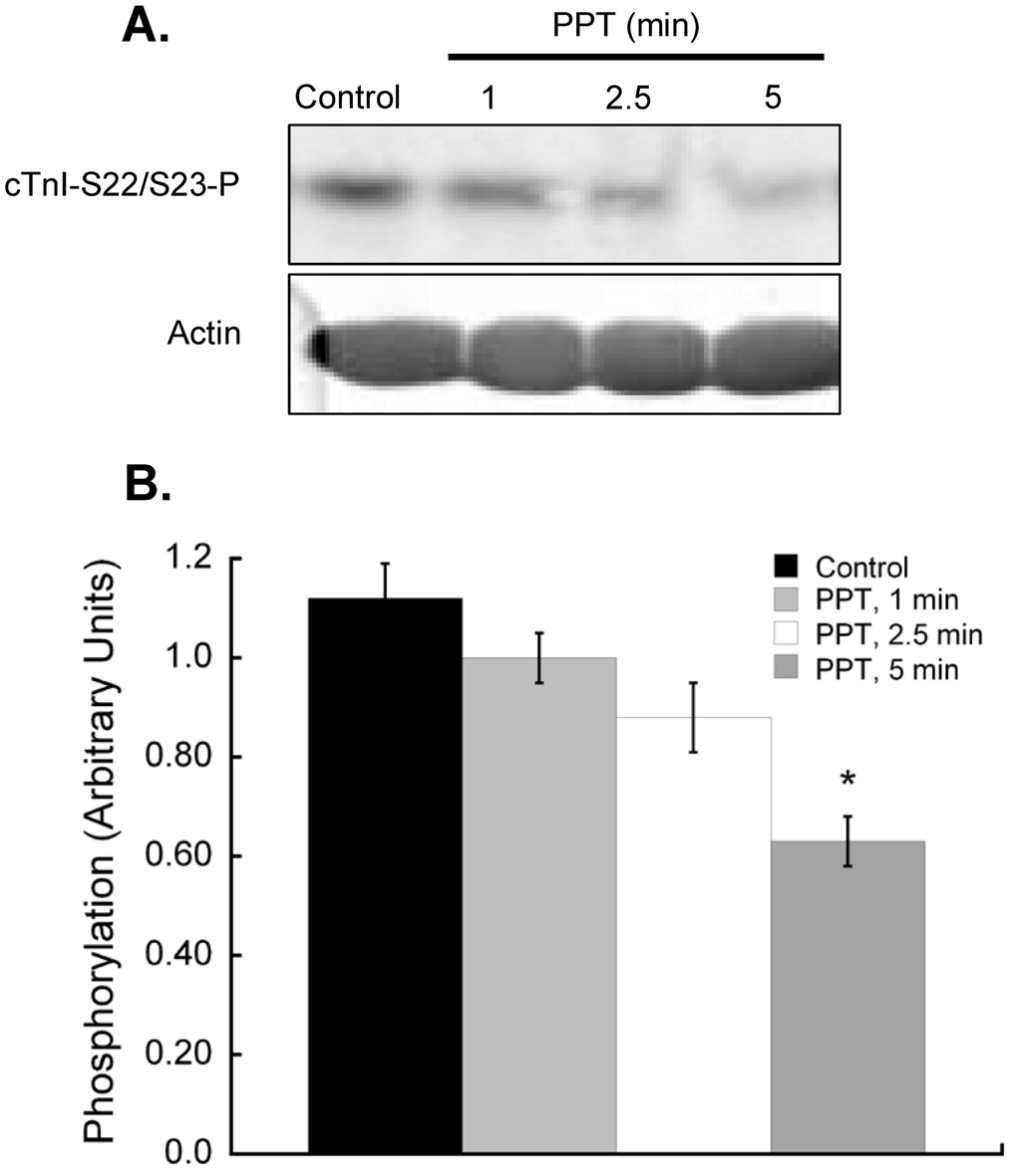
Estrogen receptor-α-specific activation decreases phosphorylation of cardiac myofilament troponin I at serines 22/23. Hearts were perfused with 100 nM PPT for 1, 2.5 or 5 minutes. Isolated cardiac myofilaments were separated by SDS-PAGE on a 10% gel. A. Composite blot of cTnI phosphorylation (serines 22/23) as measured by immunoblot analysis and normalized to total actin. B. cTnI phosphorylation (serines 22/23) decreased with PPT treatment as compared to vehicle treated controls at 5 min. N = 4.*P<0.05 vs Control.

**Figure 4:**
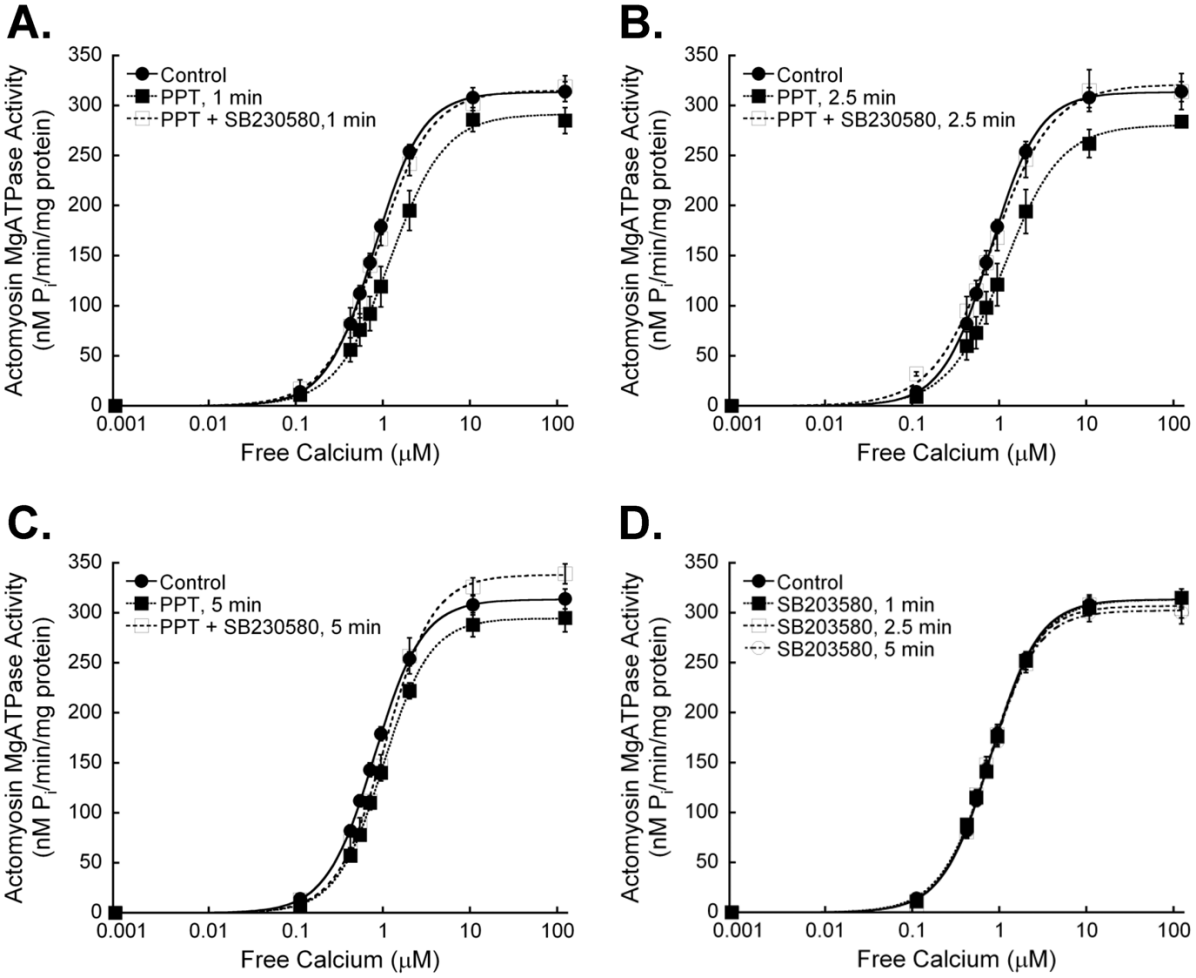
Inhibition of p38 MAPK blocks the effects of estrogen receptor-α stimulation on cardiac myofilament activation. Hearts were perfused with an inhibitor of p38 MAPK (SB203580, 1 µM) 5 min prior to and during perfusion with 100 nM PPT. **A.** 1 min PPT. **B.** 2.5 min PPT. **C.** 5 min PPT. Inhibition of p38 MAPK blocked the effects of PPT on actomyosin MgATPase activity at all time points. **D.** Treatment with SB203580 had no effect on myofilament activation at all timepoints. N = 5 for all groups.

**Table 2:** Antagonism of the effects of ERα activation on cardiac myofilament function with a p38 MAPK inhibitor.

		PPT (100 nM) + SB SB203580		
	Control	1 min	2.5 min	5 min
Maximum ATPase	314±10	320±20	314±18	339±10
EC_50_	0.81±0.03	0.90±0.10	0.85±0.03	1.09±0.05*
Hill	1.67±0.08	1.45±0.30	1.29±0.04	1.63±0.23

**Key:** Maximum ATPase, actomyosin MgATPase at maximally activating calcium, nM Pi/min/mg protein; EC_50_, free calcium (µM) required to activate actomyosin MgATPase at 50% of maximum; Hill, Hill coefficient. *P<0.05 vs control.

**Figure 5:**
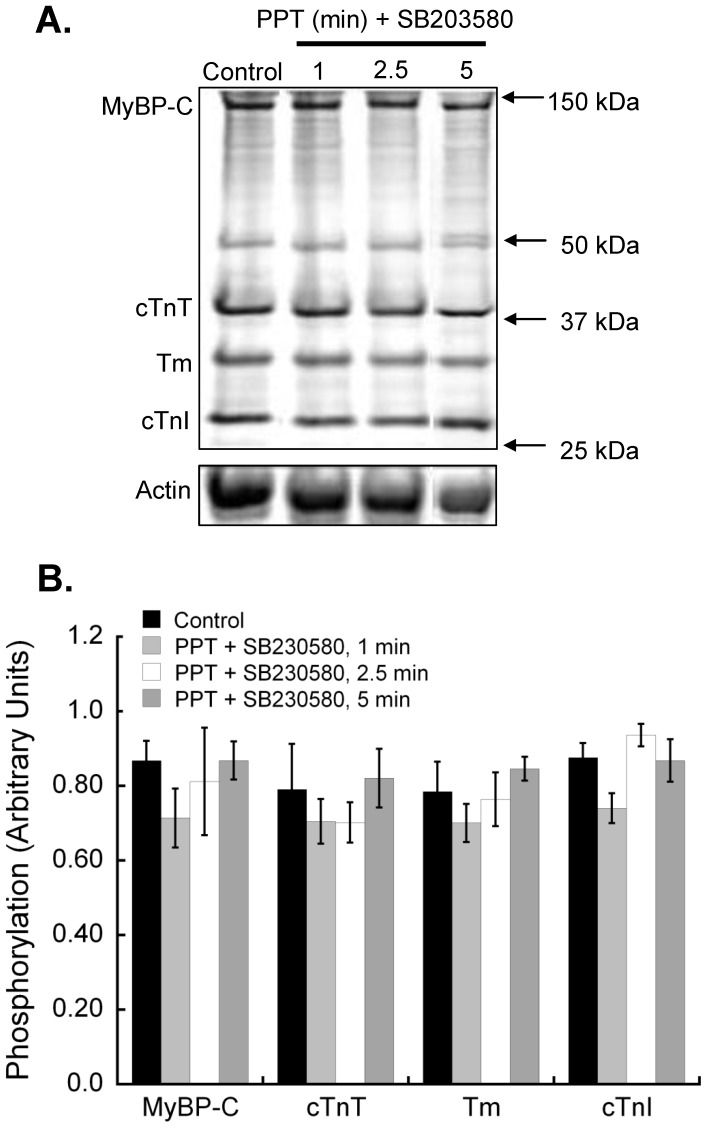
Inhibition of p38 MAPK does not alter the effects of estrogen receptor-α activation on myofilament protein phosphorylation. Hearts were perfused with the p38 MAPK inhibitor SB203580 (1 µM) for 5 min before and during PPT (100 nM) treatment. Hearts were exposed to PPT for up to 5 min and cardiac myofilament isolated. **A.** Isolated cardiac myofilaments were separated by SDS-PAGE on a 12% gel. Gels were stained with Pro-Q Diamond Phosphoprotein gel stain to assess global phosphorylation. Equal protein loading was confirmed by Coomassie staining. The actin band from Coomassie stained gels is shown as representative of protein loading. **B.** Analysis of gels for global phosphorylation of myosin-binding protein C (MyBP-C), troponin T (cTnT), tropomyosin (Tm), and troponin I (cTnI) revealed no significant changes in phosphorylation status of any myofilament protein. N = 5 for all groups.

**Figure 6:**
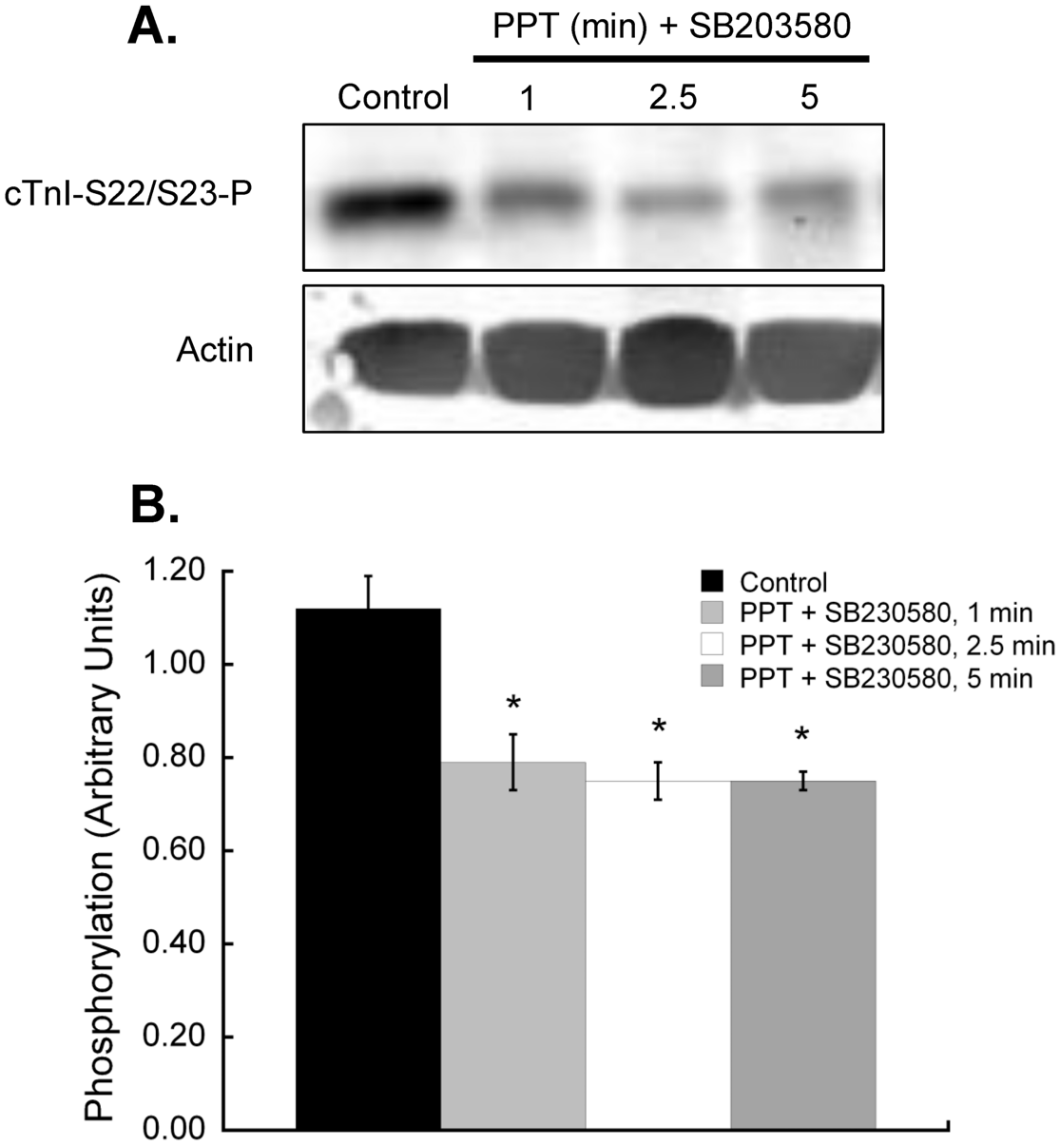
Inhibition of p38 MAPK enhances the dephosphorylation of cardiac myofilament troponin I at serines 22/23 with estrogen receptor-α-specific activation. Hearts were perfused with the p38 MAPK inhibitor SB203580 (1 µM) for 5 min, followed by treatment with 100 nM PPT for 1, 2.5 or 5 minutes in the presence of SB203580. Isolated cardiac myofilaments were separated by SDS-PAGE on a 10% gel. **A.** Composite blot of cTnI phosphorylation (serines 22/23) as measured by immunoblot analysis and normalized to total actin. **B.** cTnI phosphorylation at serines 22/23 was significantly less than control levels at all treatment times. N = 4.*P<0.05 vs Control.

## Methods

### Animal Care

Female C57Bl6 mice were obtained from Charles River Laboratories (City, PQ, Canada). All animals were cared for in accordance with the principles and guidelines provided by the Animal Care Committee at the University of Guelph.

### Heart Removal and Langendorff Perfusion

Hearts were excised from mice following euthanasia by CO_2_ inhalation, and rinsed in ice-cold saline. The aorta was cannulated and hearts were perfused at 80 mmHg with oxygenated (95% O_2_/5% CO_2_) Krebs-Henseleit buffer (pH 7.4). A balloon attached to a pressure transducer was inserted into the left ventricle via the left atrium and inflated to give an end diastolic pressure of <5 mmHg. To activate ERα, some hearts were perfused with 4,4′,4′′-(4-Propyl-[1H]-pyrazole-1,3,5-triyl) trisphenol (PPT, ERα agonist; 100 nM) for up to 5 min. This concentration of PPT maximally activates ERα without stimulating ERβ [20], and the non-genomic actions of ERα activation reach a sustained peak at 5 min [21]. Confirmation of the ERα-specific effects of PPT was done by perfusing hearts with the ERα antagonist methyl-piperidino-pyrazole (MPP, 1 µM) for 5 min before and during PPT exposure. To test for the involvement of second messengers, hearts were perfused with SB203580 (p38 MAPK antagonist; 1 µM) for five minutes prior to and during PPT perfusion. Agonist/antagonist perfusion was done by introducing drugs into the perfusate just above the canula. Following treatment, hearts were snap-frozen in liquid nitrogen and stored at -80°C until use.

### Myofilament Isolation

Myofilaments were isolated from ventricles as described previously [22], [23], [24]. Briefly, hearts were sectioned and homogenized in ice cold Standard Buffer. The homogenate was centrifuged at 14,100 g for 15 min at 4°C. Pellets were dissolved in Skinning Buffer containing 1% Triton X-100, and shaken for 45 min at 4°C before centrifugation at 1,100×g for 15 min. Resulting pellets were washed 3 times in ice-cold Standard Buffer. Isolated myofilaments were used immediately for assay of myofilament ATPase activity, or were aliquoted and frozen at -80°C for gel electrophoresis.

### Actomyosin Mg^2+^ATPase Activity

Actomyosin Mg^2+^ATPase activity was measured using a modified Carter assay as previously published [24]. Reaction buffers containing varying concentrations of free calcium were created using combinations of Activating and Relaxing buffers, and free calcium was calculated using MaxChelator software [25]. Isolated myofilaments (50 µg) were incubated in reaction buffers for 5 min at 32°C, and reactions quenched with ice cold 10% trichloroacetic acid. Inorganic phosphate (Pi) production was used as a measure of ATP consumption and cross-bridge cycling. Pi was measured by adding an equal volume of 0.5% FeSO_4_ and 0.5% ammonium molybdate in 0.5 M H_2_SO_4_, and reading the absorbance of each solution at 630 nm.

### Myofilament Protein Phosphorylation

Myofilament proteins (40 µg) were separated on a 12% SDS-polyacrylamide gel. Gels were fixed in 50% methanol/10% acetic acid at room temperature overnight and stained with Pro-Q Phosphoprotein Diamond Stain (Molecular Probes, Eugene, OR, U.S.A.) according to the manufacturer’s instructions. Imaging was done using a Typhoon gel scanner (GE Healthcare, Baie Quebec). Densitometric analysis was performed on bands representing cardiac myosin-binding protein C (MyBP-C), troponin T (TnT), tropomyosin (Tm) and troponin I (TnI) using ImageJ software (NIH, Bethesda, MD, U.S.A.). Protein loading was determined by Coomassie staining and phosphorylation measurements were normalized to protein load.

### Immunoblotting

Homogenized myocardium (150 µg) was separated on a 10% SDS-polyacrylamide gel and transferred to nitrocellulose membrane (3 h, 100 V, room temperature). Membranes were blocked with 5% bovine serum albumin (phospho-p38 MAPK) or 5% dry milk powder (total p38 MAPK) and probed overnight at 4°C with antibodies against phosphorylated p38 MAPK (1∶500) or total p38 MAPK (1∶1000) (Santa Cruz Biotechnology, Inc., Santa Cruz, CA, USA). Secondary antibodies (1∶5000) were conjugated to horseradish peroxidase (Sigma Aldrich, Oakville, Ontario, Canada). Bands were detected using Western Lightning (PerkinElmer Life and Analytical Sciences, Woodbridge, Ontario, Canada). Densitometric analysis was performed using Image J software, and the ratio of phosphorylated p38 MAPK to total p38 MAPK was calculated. Phosphorylation of cTnI at serines 23 and 24 (S23/S24) was assessed in a similar manner, except that 40 µg of isolated myofilaments were separated in a 12% SDS-polyacrylamide gel.

### Solutions

Krebs-Henseleit (KH) Buffer contained 118.5 mM NaCl, 26.6 mM NaHCO_3_, 10.0 mM D-(+) Glucose, 5.0 mM KCl, 2.0 mM NaH_2_PO_4_, 1.2 mM MgSO_4_ and 0.2 mM CaCl_2_. Standard Buffer contained 60 mM KCl, 2 mM MgCl_2_, 30 mM Imidazole (pH 7.0). Skinning Buffer contained 10 mM EGTA, 8.2 mM MgCl_2_, 14.4 mM KCl, 60 mM Imidazole (pH 7.0), 5.5 mM ATP, 12 mM creatine phosphate, 10 U/mL bovine creatine phosphokinase. Activating buffer contained 23.5 mM KCl, 5 mM MgCl_2_, 3.2 mM ATP, 2 mM EGTA, 20 mM Imidazole (pH 7.0), 2.2 mM CaCl_2_. Relaxing buffer contained 26 mM KCl, 5.1 mM MgCl_2_, 3.2 mM ATP, 2 mM EGTA, 20 mM Imidazole (pH 7.0), 4.9 µM CaCl_2_. All solutions (except KH buffer) contained protease inhibitors (0.2 mM PMSF, 0.1 mM Leupeptin and 0.1 mM Benzamidine) added just prior to use.

### Statistical Analysis

All values are presented as mean ± SEM. Statistical analysis was carried out using one-way ANOVA and a post-hoc Dunnet’s t-test (actomyosin ATPase data). Phosphorylation and immunoblot data were analyzed using Fisher’s Least Significant Difference (LSD) test. Values of P ≤ 0.05 were accepted as statistically significant.

**Figure 7:**
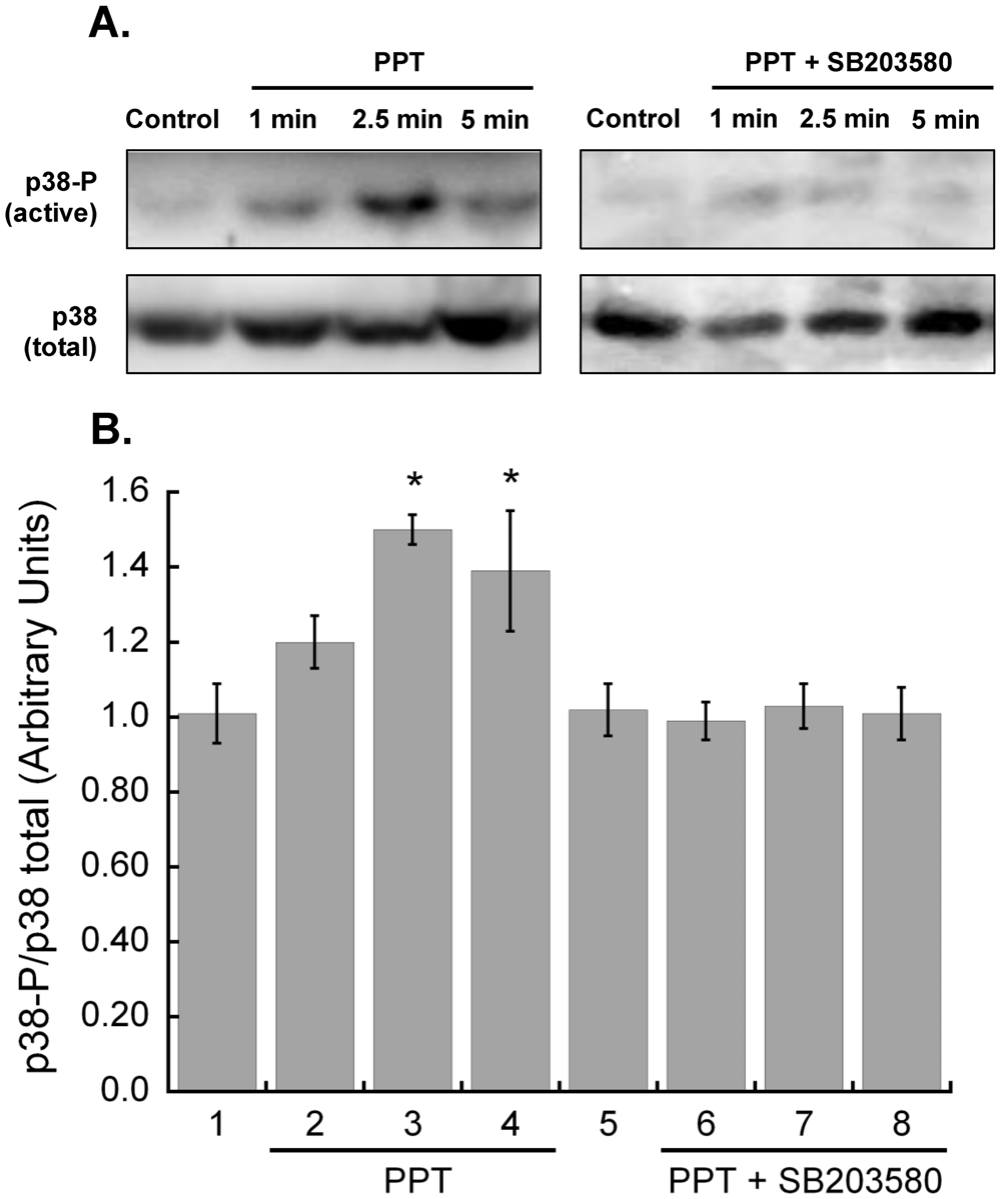
Acute stimulation of estrogen receptor-α activates p38 MAPK. Hearts were perfused with 100 nM PPT for 1, 2.5 or 5 minutes. **A.** p38 activation was assessed with immunoblot analysis of myocardium homogenates with an antibody against phosphorylated p38, and normalized to total p38. **B.** p38 activity was significantly increased following 2.5 minutes of ERα activation (N = 4, p<0.05) and remained elevated at 5 min. N = 4. SB203580 (1 µM) inhibited the PPT-dependent increase in phosphorylated p38 at all time points (N = 4 for all time points). *P<0.05 vs Control.

**Figure 8:**
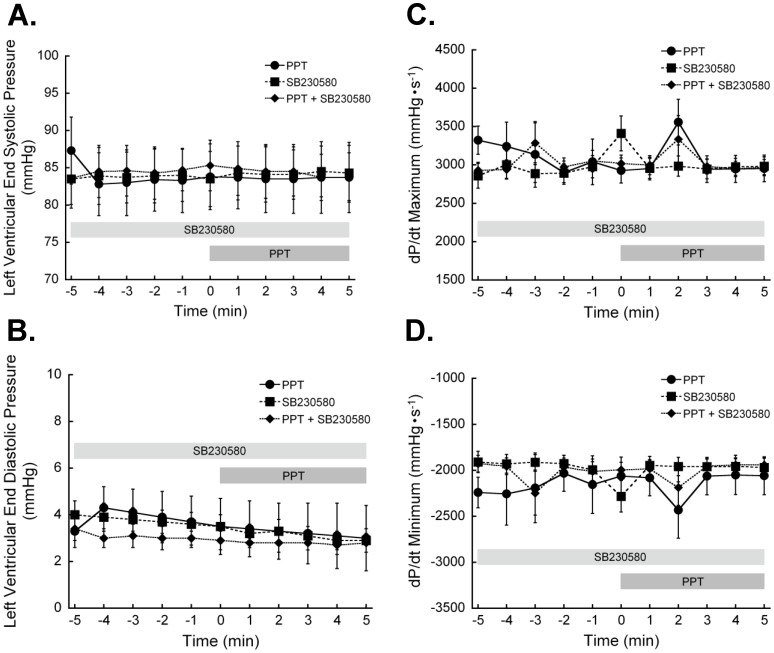
Estrogen receptor-α activation does not alter whole heart function. Hearts were perfused on a Langendorff apparatus to establish baseline pressures. The final 5 min (-5 to 0 min) before PPT treatment were taken as the baseline. Some hearts were treated with 1 µM SB203580 to inhibit p38 MAPK. PPT treatment had no significant effect on **A.** left ventricular end systolic or **B.** diastolic pressure. Neither **C.** rate of pressure development nor **D.** rate of relaxation were altered by PPT. p38 MAPK inhibition with SB203580 did not differ from controls in any parameter. (N = 6 for all time points). *P<0.05 vs Control.

## Results

### Acute Activation of ERα Modifies Cardiac Myofilament Activation

To determine whether myofilament activation can be altered through rapid, nongenomic ER signaling, we examined myofilament function of murine hearts following acute ERα activation with the selective ligand PPT. Maximum actomyosin MgATPase activity tended to be reduced following 1, 2.5 or 5 minutes of PPT treatment, but did not reach statistical significance (Figure 1, Table 1). Myofilament calcium sensitivity decreased significantly with ERα activation, as evidenced by the reduced activation at submaximal calcium levels and the increasing EC_50_ values. The ERα antagonist MPP abolished the effects of PPT, confirming the specificity of PPT for ERα. Together these results indicate that acute ERα activation rapidly decreases myofilament cross-bridge cycling and sensitivity to calcium.

### Acute ERα Activation does not Alter Global Phosphorylation of Myofilament Proteins

Changes in the phosphorylation status of myofilament proteins allow for the rapid regulation of cardiac myofilament dynamics. We sought to determine if the changes in myofilament function following acute myocardial ERα stimulation were associated with alterations in cardiac myofilament protein phosphorylation. Interestingly, there were no significant changes in the phosphorylation of MyBP-C, cTnT, Tm or cTnI following PPT treatment at various time points (Figure 2).

Cardiac myofilament proteins each contain multiple phosphorylation sites that can be individually targeted by intracellular signaling molecules. The phosphorylation of S23/S24 in the N-terminal region of murine cTnI is well known to decrease myofilament calcium sensitivity. To determine if there were site-specific changes in cTnI phosphorylation following ERα activation, we probed myofilament preparations with an antibody that recognizes the phosphorylated S23/S24 form of cTnI. Our data show that S23/S24 phosphorylation is reduced following PPT treatment, although statistical significance was only seen at 5 min (Figure 3).

### p38 MAPK Inhibition Prevents Myofilament Regulation by Rapid ERα Signaling

To establish if rapid ERα-associated myofilament changes are mediated through p38 MAPK, we perfused hearts with a p38 MAPK-specific inhibitor prior to and during PPT perfusion. The p38 MAPK inhibitor SB203580 blocked ERα-dependant effects on cardiac myofilament function, except for the change in EC_50_ at 5 min (Figure 4, Table 2). SB203580 had no effect on myofilament function when administered alone. Inhibition of p38 MAPK did not reveal any changes in global myofilament protein phosphorylation (Figure 5), nor did it stop the ER-α-dependent decrease in cTnI S23/S24 phosphorylation (Figure 6). In fact, the dephosphorylation of cTnI S23/S24 showed a significant decrease at 2.5 and 5 min of PPT treatment, suggesting that p38 MAPK attenuated the decline in phosphorylation at 2.5 min.

### Acute ERα Signaling Increases Activation of p38 MAPK

To confirm p38 MAPK involvement in acute ERα signaling, we examined the ratio of phosphorylated p38 MAPK to total p38 MAPK through immunoblot analysis. PPT increased phosphorylated p38 MAPK at all time points, and was significantly higher than control following 2.5 and 5 minutes of ERα activation (Figure 7). p38 MAPK inhibition with SB203580 abolished the increase in phosphorylated p38 MAPK.

### ERα-dependent Changes in Myofilament Activation does not Alter Myocardial Function

Hearts were perfused with 100 nM PPT in the presence and absence of SB203580, and whole heart function measured throughout the treatment period. ERα activation did not significantly impact left ventricular pressure development at any time of treatment (Figure 8). Inhibition of p38 MAPK did not reveal any significant alterations in myocardial contractile performance.

## Discussion

Estrogen signaling in the heart is thought to be cardioprotective, although this area is the subject of much debate. The mechanisms by which estrogen mediates its myocardial effects have not yet been fully elucidated, and the complexity of estrogen signaling may contribute to the discrepant results of studies examining the cardiovascular effects of hormone replacement therapy. Estrogen works through both genomic and nongenomic mechanisms, and functions by activating at least two receptor isoforms, ERα and ERβ. The various subtypes of ER exhibit different distribution patterns in cardiomyocytes and have distinct functional roles in a number of tissues. Identifying the effects and mechanisms of action linked to each ER isoform in the heart is crucial to fully understanding how estrogens regulate myocardial function. In this study we present the first data showing that the cardiac myofilaments are a target of acute ERα activation, and that stimulation of ERα decreases myofilament activity through a p38 MAPK-dependent pathway.

Few studies have explored the effects of estrogen on cardiac myofilaments. Previous studies using ovariectomy (OVX) as a model of menopause in pre-pubescent [26] and post-pubescent [27] female rats have shown that the OVX-associated depletion of E2 results in the suppression of maximum myofilament ATPase activity a hypersensitivity of the myofilaments to calcium [18]. Administration of E2 reverses these effects and restores myofilament function [19], [27]. Combined these studies suggest that E2 decreases myofilament calcium sensitivity. Consistent with these studies, we found that the specific activation of ERα similarly depresses the myofilament response to calcium.

Estrogens acutely reduce myocardial contractility, a functional effect that is due at least in part to decreased calcium transients [28]. Interestingly, Ullrich et al [29] reported that the effects of estrogens on myocardial activation may be mediated through estrogen receptor-independent mechanisms. These findings suggest that understanding the roles of the various estrogen receptors may necessitate using pharmacological agents that specifically target the receptors of interest. Our results show that left ventricular contractility is not significantly affected by ERα activation, but that myofilament calcium sensitivity is reduced. The lack of any effect of ERα activation on myocardial contractility is consistent with Filice et al [30] who reported similar findings. The combination decreased myofilament calcium sensitivity with no change in myocardial contractility leads to the suggestion that acute ERα stimulation with PPT may increase intracellular calcium transients, off-setting the reduction in myofilament activation. Although this hypothesis is in contradiction to earlier studies showing a reduction in calcium transients with acute estrogen exposure [28], the difference may be explained by the non-specific ER activation in previous studies.

Previous studies have noted only a marginal effect of estrogen on p38 MAPK activation. In one study, 5 minute or longer treatment of adult cardiomyocytes with 1 nM E2 failed to show a significant increase in p38 MAPK phosphorylation either through Western blot analysis or phosphorylation assay, whereas activation of ERK1/2 and JNK was observed [17]. Furthermore, a comparison of female wild-type and ERα knockout mice showed no difference in p38 MAPK activation, but an increase in protective ERK1/2 and a decrease in JNK activation during ischemia [31]. This discrepancy may be due to differences in experimental protocols between our study and those mentioned. Whereas E2 activates ERα, ERβ and potentially the GPR30, our work looks at specific ERα activation. Cross-talk between multiple ER may result in the activation of different MAPK signaling pathways. Moreover, systemic and lifelong ERα knockout [31] may alter signaling pathways as a compensatory response.

Changes in circulating estrogens tend to occur over prolonged periods of time. The rapid effects examined in the current study may not necessarily reflect physiological events in which circulating estrogen levels fluctuate quickly. However, there is evidence to suggest a local, acute, cardiac metabolic pathway, making this a feasible possibility [32]. Where our data do provide applicable insight is the examination of acute signaling changes associated with ERα activation and potentially cardioprotective signaling cascades. Cardioprotection with ERα activation occurs on the timescale of minutes, similar to the timeline used in our studies, and the intracellular mechanisms of action are not fully understood [15]. Our previous work suggesting a role for myofilament modifications in cardioprotection [22], [23] validates the exploration of ERα-dependent regulation of contractile regulation, and generates new information about the intracellular mechanisms of action associated with ERα stimulation.

The current study presents insight into the myocardial effects of acute ERα activation and the intracellular pathways by which this receptor may regulate nongenomic function and myofilament activation. We found that short-duration treatment of myocardium with the ERα isoform-selective agonist PPT results in the activation of p38 MAPK, and a decrease in myofilament calcium sensitivity. These changes were not associated with any observable alteration in total myofilament protein phosphorylation, but variations in the pattern of cTnI phosphorylation were detected. The complex and controversial nature of cardiovascular regulation by estrogens necessitates an in-depth investigation of how ER control cardiovascular function. This includes an understanding of the role ER-isoforms play in regulating heart function and their intracellular mechanisms of action. A clear picture of how ER affect myocardial function under physiological conditions provides a fundamental basis for future investigations into the role of estrogens in cardiovascular disease, including the controversies associated with hormone replacement therapy.

## References

[1] Mendelsohn ME, Karas RH (1999) The protective effects of estrogen on the cardiovascular system. N Engl J Med 340: 1801–1811. 10362825 10.1056/NEJM199906103402306

[2] Stampfer MJ, Colditz GA (1991) Estrogen replacement therapy and coronary heart disease: a quantitative assessment of the epidemiologic evidence. Prev Med 20: 47–63. 1826173 10.1016/0091-7435(91)90006-p

[3] Donaldson C, Eder S, Baker C, Aronovitz MJ, Weiss AD, et al (2009) Estrogen attenuates left ventricular and cardiomyocyte hypertrophy by an estrogen receptor-dependent pathway that increases calcineurin degradation. Circ Res 104: 265–275, 211p following 275. 10.1161/CIRCRESAHA.108.190397 PMC4427027 19074476

[4] Gardner JD, Brower GL, Voloshenyuk TG, Janicki JS (2008) Cardioprotection in female rats subjected to chronic volume overload: synergistic interaction of estrogen and phytoestrogens. Am J Physiol Heart Circ Physiol 294: H198–204. 17965290 10.1152/ajpheart.00281.2007

[5] Pedram A, Razandi M, Lubahn D, Liu J, Vannan M, et al (2008) Estrogen inhibits cardiac hypertrophy: role of estrogen receptor-beta to inhibit calcineurin. Endocrinology 149: 3361–3369. 18372323 10.1210/en.2008-0133 PMC2453079

[6] Anderson GL, Limacher M, Assaf AR, Bassford T, Beresford SA, et al (2004) Effects of conjugated equine estrogen in postmenopausal women with hysterectomy: the Women’s Health Initiative randomized controlled trial. JAMA 291: 1701–1712. 15082697 10.1001/jama.291.14.1701

[7] Hulley S, Grady D, Bush T, Furberg C, Herrington D, et al (1998) Randomized trial of estrogen plus progestin for secondary prevention of coronary heart disease in postmenopausal women. Heart and Estrogen/progestin Replacement Study (HERS) Research Group. JAMA 280: 605–613. 9718051 10.1001/jama.280.7.605

[8] Manson JE, Hsia J, Johnson KC, Rossouw JE, Assaf AR, et al (2003) Estrogen plus progestin and the risk of coronary heart disease. N Engl J Med 349: 523–534. 12904517 10.1056/NEJMoa030808

[9] Dubey RK, Imthurn B, Barton M, Jackson EK (2005) Vascular consequences of menopause and hormone therapy: importance of timing of treatment and type of estrogen. Cardiovasc Res 66: 295–306. 15820198 10.1016/j.cardiores.2004.12.012

[10] Grohe C, Kahlert S, Lobbert K, Vetter H (1998) Expression of oestrogen receptor alpha and beta in rat heart: role of local oestrogen synthesis. J Endocrinol 156: R1–7. 9518889 10.1677/joe.0.156r001

[11] Lizotte E, Grandy SA, Tremblay A, Allen BG, Fiset C (2009) Expression, distribution and regulation of sex steroid hormone receptors in mouse heart. Cell Physiol Biochem 23: 75–86. 19255502 10.1159/000204096

[12] Deschamps AM, Murphy E (2009) Activation of a novel estrogen receptor, GPER, is cardioprotective in male and female rats. Am J Physiol Heart Circ Physiol 297: H1806–1813. 19717735 10.1152/ajpheart.00283.2009 PMC2781389

[13] Ropero AB, Eghbali M, Minosyan TY, Tang G, Toro L, et al (2006) Heart estrogen receptor alpha: distinct membrane and nuclear distribution patterns and regulation by estrogen. J Mol Cell Cardiol 41: 496–510. 16876190 10.1016/j.yjmcc.2006.05.022

[14] Booth EA, Obeid NR, Lucchesi BR (2005) Activation of estrogen receptor-alpha protects the in vivo rabbit heart from ischemia-reperfusion injury. Am J Physiol Heart Circ Physiol 289: H2039–2047. 15994857 10.1152/ajpheart.00479.2005

[15] Novotny JL, Simpson AM, Tomicek NJ, Lancaster TS, Korzick DH (2009) Rapid estrogen receptor-alpha activation improves ischemic tolerance in aged female rats through a novel protein kinase C epsilon-dependent mechanism. Endocrinology 150: 889–896. 19176323 10.1210/en.2008-0708

[16] Hsu JT, Hsien YC, Kan WH, Chen JG, Choudhry MA, et al (2007) Role of p36 mitogen-activated protein kinase pathway in estrogen-mediated cardioprotection following trauma-hemorrhage. Am J Physiol Heart Circ Physiol 292: H2982–H2987. 17293487 10.1152/ajpheart.01303.2006

[17] Nuedling S, Kahlert S, Loebbert K, Meyer R, Vetter H, et al (1999) Differential effects of 17beta-estradiol on mitogen-activated protein kinase pathways in rat cardiomyocytes. FEBS Lett 454: 271–276. 10431821 10.1016/s0014-5793(99)00816-9

[18] Wattanapermpool J (1998) Increase in calcium responsiveness of cardiac myofilament activation in ovariectomized rats. Life Sci 63: 955–964. 9747896 10.1016/s0024-3205(98)00353-1

[19] Wattanapermpool J, Riabroy T, Preawnim S (2000) Estrogen supplement prevents the calcium hypersensitivity of cardiac myofilaments in ovariectomized rats. Life Sci 66: 533–543. 10794070 10.1016/s0024-3205(99)00623-2

[20] Escande A, Pillon A, Servant N, Cravedi JP, Larrea F, et al (2006) Evaluation of ligand selectivity using reporter cell lines stably expressing estrogen receptor alpha or beta. Biochem Pharmacol 71: 1459–1469. 16554039 10.1016/j.bcp.2006.02.002

[21] Chen Z, Yuhanna IS, Galcheva-Gargova Z, Karas RH, Mendelsohn ME, et al (1999) Estrogen receptor alpha mediates the nongenomic activation of endothelial nitric oxide synthase by estrogen. J Clin Invest 103: 401–406. 9927501 10.1172/JCI5347 PMC407904

[22] Pyle WG, Chen Y, Hofmann PA (2003) Cardioprotection through a PKC-dependent decrease in myofilament ATPase. Am J Physiol Heart Circ Physiol 285: H1220–1228. 12763745 10.1152/ajpheart.00076.2003

[23] Pyle WG, Smith TD, Hofmann PA (2000) Cardioprotection with kappa-opioid receptor stimulation is associated with a slowing of cross-bridge cycling. Am J Physiol Heart Circ Physiol 279: H1941–1948. 11009483 10.1152/ajpheart.2000.279.4.H1941

[24] Yang F, Aiello DL, Pyle WG (2008) Cardiac myofilament regulation by protein phosphatase type 1alpha and CapZ. Biochem Cell Biol 86: 70–78. 18364747 10.1139/o07-150

[25] Patton C, Thompson S, Epel D (2004) Some precautions in using chelators to buffer metals in biological solutions. Cell Calcium 35: 427–431. 15003852 10.1016/j.ceca.2003.10.006

[26] Schaible TF, Malhotra A, Ciambrone G, Scheuer J (1984) The effects of gonadectomy on left ventricular function and cardiac contractile proteins in male and female rats. Circ Res 54: 38–49. 6229365 10.1161/01.res.54.1.38

[27] Scheuer J, Malhotra A, Schaible TF, Capasso J (1987) Effects of gonadectomy and hormonal replacement on rat hearts. Circ Res 61: 12–19. 2955949 10.1161/01.res.61.1.12

[28] Jiang C, Poole-Wilson PA, Sarrel PM, Mochizuki S, Collins P, et al (1992) Effect of 17 beta-oestradiol on contraction, Ca2+ current and intracellular free Ca2+ in guinea-pig isolated cardiac myocytes. Br J Pharmacol 106: 739–745. 1504758 10.1111/j.1476-5381.1992.tb14403.x PMC1907543

[29] Ullrich ND, Krust A, Collins P, MacLeod KT (2008) Genomic deletion of estrogen receptors ERalpha and ERbeta does not alter estrogen-mediated inhibition of Ca2+ influx and contraction in murine cardiomyocytes. Am J Physiol Heart Circ Physiol 294: H2421–2427. 18441199 10.1152/ajpheart.01225.2007

[30] Filice E, Angelone T, De Francesco EM, Pellegrino D, Maggiolini M, et al (2011) Crucial role of phospholamban phosphorylation and S-nitrosylation in the negative lusitropism induced by 17beta-estradiol in the male rat heart. Cell Physiol Biochem 28: 41–52. 21865847 10.1159/000331712

[31] Wang M, Crisostomo P, Wairiuko GM, Meldrum DR (2006) Estrogen receptor-alpha mediates acute myocardial protection in females. Am J Physiol Heart Circ Physiol 290: H2204–2209. 16415070 10.1152/ajpheart.01219.2005

[32] Bell JR, Mellor KM, Wollermann AC, Ip WT, Reichelt ME, et al (2011) Aromatase deficiency confers paradoxical postischemic cardioprotection. Endocrinology 152: 4937–4947. 22028441 10.1210/en.2011-1212

